# Molecular characterization of avian pathogenic *Escherichia coli* isolates from broiler farms in Northern Palestinian territories

**DOI:** 10.14202/vetworld.2024.2865-2879

**Published:** 2024-12-19

**Authors:** Ghaleb Adwan, Sameh Abuseir, Ghadeer Omar, Mahmoud Albzour

**Affiliations:** 1 Department of Biology and Biotechnology, Molecular Microbiology/Virology, An-Najah National University, Nablus, Palestinian Territories; 2 Department of Veterinary Medicine, Faculty of Agriculture and Veterinary Medicine, An-Najah National University, Nablus, Palestinian Territories; 3 Department of Natural Sciences, Faculty of Graduate Studies, An-Najah National University, Nablus, Palestinian Territories

**Keywords:** antibiotic resistance, avian pathogenic *Escherichia coli*, colibacillosis, Palestinian territories, phylogenetic group, virulence factors

## Abstract

**Background and Aim:**

Colibacillosis is caused by avian pathogenic *Escherichia coli* (APEC), which results in significant losses for the poultry sector. It has zoonotic potential and acts as a source of antibiotic resistance and virulence genes for other *E. coli*. This study aimed to assess phylogenetic groups, virulence factors, and resistance phenotypes of APEC strains isolated from broiler farms in Northern Palestine.

**Materials and Methods:**

A total of 65 APEC isolates were recovered from diseased chickens with typical colibacillosis symptoms from broiler farms located in the northern region of Palestine from May to July 2024. Strains were identified using classical and molecular techniques. Antibiotic resistance was detected using the disk diffusion method. Phylotyping and virulence genotyping of the APEC isolates were performed using a polymerase chain reaction (PCR).

**Results:**

This study showed a high detection rate of APEC strains (100%) in chickens. The most APEC strains, 56/65 (86.2%), belonged to group D. Other strains were assigned to groups B2 (5/65, 7.7%), B1 (3/65, 4.6%), and A (1/65, 1.5%). Antibiotic resistance ranged from 27.7% for Polymyxin E (colistin) to 100% for Amoxicillin. Polymyxin E (colistin) and fosfomycin are the most effective drugs. The most common virulence gene was *iroN*, which was detected in 61 isolates (93.8%). The APEC strains in Palestine exhibit a wide variety of resistance patterns and genetic variations.

**Conclusion:**

Controlling APEC infections is essential for public health, especially when APEC isolates can pass on resistance and virulence genes to dangerous bacteria such as *E. coli* that are particular to humans. It is essential to understand APEC pathogenesis, antimicrobial therapy, and the development of measures to control colibacillosis.

## Introduction

*Escherichia coli* belongs to the *Enterobacteriaceae* family of commensal bacteria that occurs frequently in the digestive tracts of warm-blooded animals, where it symbiotically participates in the production of vitamins and digestion. There are currently approximately 160 serological forms of *E. coli*, in addition to 171 somatic (O), 55 flagellar (H), and 80 capsular (K) antigens. Three primary *E. coli* strains have been identified: extraintestinal pathogenic *E. col* (ExPEC), intestinal pathogenic or diarrheagenic *E. coli* strains (DEC), and commensal strains. One of the most important bacteria that may lead to diarrhea is DEC, which can also cause gastroenteritis. Hemolytic–uremic syndrome, meningitis, inflammation of the meninges, sepsis, surgical site infection, urinary tract infection, and hospital-acquired pneumonia are all associated with ExPEC [1]. Avian pathogenic *Escherichia coli* (APEC), a subpathotype of ExPEC, has emerged as a primary pathogen in avian hosts, causing avian colibacillosis, a syndrome characterized by a variety of localized and generalized infections [2]. The most common lesions are omphalitis, cellulitis, pericarditis, perihepatitis, airsacculitis, pericarditis, egg peritonitis, salpingitis, coligranuloma, and systemic infections. Numerous virulence factors (VFs) that are encoded on plasmids, bacteriophages, or the bacterial chromosomes inside pathogenicity islands (PAIs) and other mobile elements are present in *E. coli* strains that cause illnesses [3]. Pathogenic *E. coli* strains acquire virulence operons from non-pathogenic strains through chromosomal or extrachromosomal transfer [4]. Several studies have shown that some VFs encoded by diverse genes enhance the pathogenicity of APEC, leading to colibacillosis and growth in the tissues of broilers [5, 6]. The traditional diagnostic technique used in laboratories to identify *E. coli* linked to colibacillosis involves serotyping APEC strains based on the identification of somatic O antigens using a polymerase chain reaction (PCR) or antisera [2]. The most commonly used approach for identifying and classifying the causative agent of avian colibacillosis is the PCR-typing method, which targets 10–15 VFs and designates isolates with ≥5 virulence determinants as APECs. These isolates are obtained from hens that exhibit specific clinical signs or symptoms [3, 6–9]. Many studies have reported antimicrobial sensitivity to APEC isolates [5, 6, 8–11]. The occurrence of VFs among APEC isolates was determined [3, 5–7, 9–13]. The distribution of APEC isolates into phylogenetic groups has been demonstrated in numerous studies [3, 12–15].

Infections by APEC isolates cause a high rate of morbidity and mortality in chickens, as well as decreased production of meat and eggs, a lower rate of hatching, and a higher rate of carcasses being condemned at slaughter [3, 5, 14]. This represents economic and financial losses to the global chicken industry [3, 10]. In addition to aerosol or fecal-oral routes, contaminated food and water are common ways, in which chickens acquire the disease. Furthermore, APEC can move vertically from ill breeders to commercial day-old chicks through contaminated eggs. This gives the poultry industry a serious cause for concern. Moreover, APEC is common in all age categories of chickens (9.5%–36.7%) [3]. Some APEC strains and human ExPEC strains share common phylogenic backgrounds and similar VFs, and these strains can cause infections in humans [10, 15]. Furthermore, similarities have been observed between APEC strains in broilers and ExPEC in humans, suggesting there is a high likelihood of horizontal gene transfer between APEC and phylogenetically comparable human ExPEC strains [10]. Genomic analysis of APEC strains showed that they may be APEC strains and reservoirs for human-pathogenic ExPEC virulence genes. Ten major virulence genes (*iutA*, *iucC*, *cvaC*, *iucD*, *cvaB*, *cvaA*, *cvi*, *ompT*, *hlyF*, and *etsA*) shared by avian-associated Colicin V plasmids (ColV) in human ExPECs and APECs indicate a potential zoonotic transmission of APEC from poultry to people [16–18]. The management of avian colibacilloses can benefit the health of humans and animals. The information obtained from this study will help in the treatment of and understanding of disease progression due to the presence of VFs and genetic diversity of APEC strains that were isolated from chickens. In addition, this study will provide information on the associations between phylogenetic groups, antibiotic resistance, and VFs.

As of today, we have not found any previous studies on this topic conducted in Northern Palestine. Regarding the APEC isolates found in Northern Palestine, no information is available on their phylogenetic origin, multidrug resistance (MDR), or virulence-associated genes. Because of their various interconnections, the relationships among phylogenetic groups, antibiotic resistance, and APEC VFs are complex. This study aimed to assess phylogenetic groups, VFs, and resistance phenotypes in a collection of APEC strains isolated from chickens to clarify whether the VFs are directly connected with resistance or, instead, are dependent on a phylogenetic group distribution.

## Materials and Methods

### Ethical approval and Informed consent

This study did not require ethical approval as per the Ethics Committee of the University. However, the animals were handled carefully during the collection of the samples. Prior consent was obtained from the owner of the animal farms.

### Study period and location

The study was conducted from May to July 2024. Samples were collected from several broiler farms in the Northern Palestinian territories.

### Sample collection

In total, 65 dead chickens were brought for postmortem inspection with suspicion of colibacillosis and were diagnosed based on a pool of samples. Each pool comprised samples of the heart, liver, peritoneum, and lung from broiler farms in the northern Palestine. We collected these samples during the postmortem collection of chickens of various ages with lesions of omphalitis, cellulitis, salpingitis, egg peritonitis, airsacculitis, perihepatitis, and pericarditis. Samples were collected aseptically using a sterile cotton swab in a sterile 5 mL of tryptone soy broth (TSB: HiMedia Laboratories, India) in farms’ labaratory.

### Phenotypic *identification of E. coli*

Pre-enrichment samples in TSB were incubated for 6 h at 37°C and then subcultured onto eosin methylene blue agar (EMB agar: HiMedia Laboratories). The plates were incubated at 37°C for 18–24 h to promote bacterial growth. Green metallic sheen colonies were selected for further confirmation through biochemical tests. Gram staining was used to investigate the morphological and staining properties of the bacteria and to provide information regarding possible bacterial identification. Isolated organisms with supporting growth characteristics were identified. A variety of biochemical tests, including the catalase test, Indole test, Methyl Red (MR) test, Voges–Proskauer (VP) test, Simmons’ citrate, triple sugar iron agar (HiMedia Laboratories), and sulfide indole motility medium (HiMedia Laboratories) test, were carried out to confirm the identification of the specific *E. coli* bacteria. Further confirmation of the positive samples was performed using specific PCR primers targeting the housekeeper alkaline phosphatase (*phoA*) gene.

### Antibiotic resistance test

Using the disk diffusion method, antibiotic resistance was identified in accordance with the Clinical and Laboratory Standard Institute (CLSI) guidelines [19]. All APEC isolates were evaluated for resistance to nine distinct antibiotics (Oxoid, England) classes: aminopenicillin (amoxicillin [AX] [30 μg]), cephalosporines (ceftriaxone [CRO] [30 μg], cephalexin [CL] [30 μg], and ceftiofur [FUR] [30 μg]), tetracyclines (doxycycline [10 μg]), fluoroquinolones (ciprofloxacin [CIP] [10 μg], norfloxacin [NOR] [10 μg], and enrofloxacin [ENR] [5 μg]), aminoglycosides (gentamicin [CN] [10 μg] and neomycin [30 μg]), sulfonamides (trimethoprim/sulfamethoxazole [1.25/23.75 μg]), polymyxins (polymyxin B [PB] [10 μg]), polymyxin E (Colistin [10 μg]), amphenicols (florfenicol [FFC] [30 μg]), and phosphonic antibiotic (fosfomycin [FO] [50 μg]). A 6–8 h-old culture of the APEC isolates was swabbed into Mueller–Hinton agar (MHA: HiMedia Laboratories) plates, and then the antibiotic disks were placed on the APEC swabbed MHA plates. The plates were then incubated at 37°C for 24 h. The inhibition zones (if any) were determined according to the CLSI guidelines [19]. The isolates were classified as sensitive, intermediate, or resistant. All antimicrobial susceptibility testing experiments involved the *E. coli* (ATCC 25922) strain as a quality control strain.

### DNA extraction

Using a previously mentioned protocol, the genome of *E. coli* was isolated for PCR [20]. In short, a small number of colonies were removed from an overnight MHA plate, combined with approximately 1 mL of Tris- ethylenediaminetetraacetic acid (EDTA) buffer (Sigma Aldrich, USA) (10 mM Tris-HCl, 1 mM EDTA [pH 8]), and then centrifuged at 14,000× *g* for 5 min to pellet the cells. The pellet was resuspended in 0.5 mL of sterile distilled water and boiled for 10–15 min. The samples were then directly incubated on ice for 5–10 min. Debris was removed by centrifugation at 14,000× *g* for 5 min. The extracted DNA samples were assessed for quality and quantity using a nano-drop spectrophotometer (GenovaNano, Jenway) and then stored at –20°C until they were needed for additional DNA analysis.

### Confirmation of *E. coli* isolates using PCR

To confirm *E. coli* isolates, the amplification housekeeping gene *phoA* was selected as a target gene. The primer sequences and amplicon sizes are given in Table-1 [21]. Each PCR reaction mixture contained a total volume of 25 μL. A total volume of 12.5 μL of PCR premix containing MgCl_2_ (GoTaq® Green Master Mix, Promega, USA), 0.4 μM of each primer, 3 μL of DNA template, and a PCR distilled water was added to complete the PCR reaction mixture up to a final volume. A DNA thermal cycler (Mastercycler personal, Eppendorf, Germany) was used to amplify DNA under the following thermal conditions: initial denaturation for 3 min at 94°C followed by 30 cycles of denaturation at 94°C for 30 s, annealing at 55°C for 30 s, and extension at 72°C for 30 s, with a final extension step at 72°C for 5 min. A volume of 20 μL of the PCR product was subjected to electrophoresis on 1.5% agarose gel to identify the size of the amplified PCR fragment after staining with 0.5 μg/mL of ethidium bromide dye. A 100-bp ladder (GeneDireX, Inc. Taiwan), was used for electrophoresis. The *E. coli* (ATCC 25922) strain was used as the positive control.

**Table-1:** Primer sequences used in this study to confirm *Escherichia coli* diagnosis and phylogenetic classification of *E. coli* isolates.

Target genes	Primer sequence 5→3				Size (bp)	Pool	References
*yjaA*	yjaA F TGA AGT GTC AGG AGA CGC TG yjaA R ATG GAG AAT GCG TTC CTC AAC				211	1	[22]
*ChuA*	ChuA F GAC GAA CCA ACG GTC AGG AT ChuA R TGC CGC CAG TAC CAA AGA CA				279	1	[22]
*TspE4C2*	TspE4C2F GAG TAA TGT CGG GGC ATT CA TspE4C2R CGC GCC AAC AAA GTA TTA CG				154	1	[22]
*phoA*	phoAF: TAC, AGG; TGA, CTG; CGG, GCT; TAT, C phoA R: CTT ACC GGG CAA TAC ACT CAC TA				622	0	[21]

**Phylogroups and subgroups obtained by applying the Clermont method**							

**Gene PCR fragments**				**Phylogroup and subgroup assignment**			

***ChuA***		***yjaA***	***TspE4.C2***				

-		-	-	A (A0)			
-		+	-	A (A1)			
-		-	+	B1			
+		+	-	B2 (B22)			
+		+	+	B2 (B23)			
+		-	-	D (D1)			
+		-	+	D (D2)			

PCR=Polymerase chain reaction

### Phylotyping of APEC isolates using PCR

Using triplex PCR, the APEC strains were categorized into one of the four phylogenetic groups (A, B1, B2, and D) according to the three DNA fragments *chu*A, *yja*A, and *TspE4C2* [22]. In this study, the primer sequences and fragment sizes are presented in Table-1. According to Branger *et al*. [23], the APEC isolates could be grouped into phylogenetic groups and sub-groups based on the combination (presence or absence) of the three PCR product fragments, as shown in Table-1. The APEC isolates were categorized into phylogenetic groups and subgroups based on the existence or lack of PCR product fragments. Each PCR reaction mixture contained a total volume of 25 μL. For this PCR reaction mixture, 12.5 μL of PCR premix containing MgCl_2_ (GoTaq® Green Master Mix, Promega), 0.3 μM of each primer, 3 μL (50–100 ng) of DNA template, and a PCR distilled water was added to complete the PCR reaction mixture up to a final volume. The conditions for DNA amplification and electrophoresis are described in the previous section. Positive control strains for different phylogroup (department collection) were used [24].

### Virulence genotyping of APEC isolates using PCR

The extracted DNA was used to detect virulence genes. In total, 11 genes were detected in *E. coli* isolates from chickens with symptoms and signs of colibacillosis. The process of investigation used multiplex PCR to divide these genes into 3 pools. The detected genes were *Iss*, *tsh*, *iutA*, *iroN*, *hlyF*, *ompT*, *papGI*, *papGII*, *papGI II*, pap*C*, and *ColV*. The primer sequences for these genes, amplicon sizes, annealing temperatures, and pools are listed in Table-2 [5]. Each 25 μL PCR reaction mixture contained 3 μL (50–100 ng) of DNA template, 0.3 μM of primers, 12.5 μL of PCR premix with MgCl_2_ (GoTaq® Green Master Mix, Promega), and a PCR distilled water was added to complete the PCR reaction mixture up to a final volume. The target genes were amplified using the following PCR thermal program, which was carried out using a thermal cycler (Mastercycler personal, Eppendorf): three minutes of initial denaturation at 94°C, followed by 25 cycles of denaturation at 94°C for 30 s, annealing at 58°C for 30 s, extension at 72°C for 1 min, and final extension at 72°C for 5 min. The PCR products were analyzed by electrophoresis on a 2% agarose gel. The sizes of the PCR amplicons were determined through comparison with a 100-bp DNA ladder (GeneDireX). Positive control strains for different VFs (department collection) were used [24].

**Table-2:** Virulence gene primer sequences used in this study, their amplicon sizes, annealing temperatures, and pools [5].

Virulence gene	Description	Primer sequence 5→3	Product size (bp)	Annealing temperature	Pool
*iss*	Increased serum survival gene expression (Protectins)	iss-F AGCAACCCGAACCACTTGATG iss-F AGCATTGCCAGAGCGGCAGAA	323	58	I
*tsh*	Temperature-sensitive hemagglutinin gene (Adhesins)	tsh-F GGGAAATGACCTGAATGCTGG tsh-R CCGCTCATCAGTCAGTACCAC	420	58	I
*iutA*	Ferric aerobactin receptor gene; iron transport (Iron acquisition)	iutA-F GGCTGGACATCATGGGAACTGG iutA-F CGTCGGGAACGGGTAGAATCG	302	58	I
*iroN*	Catecholate siderophore receptor gene (Iron acquisition)	iroN-F AAGTCAAAGCAGGGGTTGCCCG iroN-R GACGCCGACATTAAGACGCAG	667	58	I
*hlyF*	Hemolysin F gene (Toxins)	hlyF-F GGCGATTTAGGCATTCCGATACTC hlyF-R ACGGGGTCGCTAGTTAAGGAG	599	58	I
*ompT*	Outer membrane protease gene (Protectins)	ompT-F ATCTAGCCGAAGAAGGAGGC ompT-R CCCGGGTCATAGTGTTCATC	559	58	I
*papGI*	Pyelonephritis-associated pili allele I (Adhesins)	papGI-F TCGTGCTCAGGTCCGGAATTT papGI-R TGGCATCCCCCAACATTATCG	461	58	II
*papGII*	Pyelonephritis-associated pili allele II (Adhesins)	papGII-F GGGATGAGCGGGCCTTTGAT papGII-R CGGGCCCCCAAGTAACTCG	190	58	II
*papGIII*	Pyelonephritis-associated pili allele III (Adhesins)	papGIII-F GGCCTGCAATGGATTTACCTGG papGIII-R CCACCAAATGACCATGCCAGAC	258	58	II
*papC*	Encoding of P pilus (Adhesins)	papC-F TGATATCACGCAGTCAGTAGC papC-R CCGGCCATATTCACATAAC	501	58	III
*cva/cvi*	Colicin V operon	ColV-F TCCAAGCGGACCCCTTATAG ColV-R CGCAGCATAGTTCCATGCT	598	58	III

### Statistical analysis

The antibiotic resistance or VF scores were calculated for each isolate as the sum of all antibiotic resistance or VF genes found in each strain [25]. This study used different statistical tests to analyze the obtained data. These tests included the Mann–Whitney U-Test (Two-tailed), T-Test Calculator for 2 Independent (Two-tailed), and Means Chi-square (χ^2^) test. Values were considered statistically significant if p < 0.05.

## Results

### Classical and molecular identification of APEC isolates

**Figure-1:**
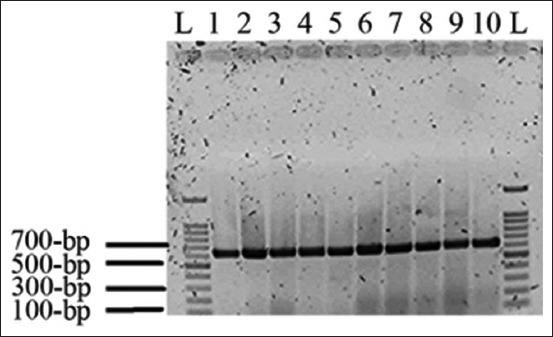
The polymerase chain reaction (PCR) product of *Escherichia coli*-specific *phoA* gene. Lane L: 100-bp ladder, lanes 1-10 the PCR product (622-bp) band of *E. coli*-specific *phoA* gene.

The colonies of the isolated strains grown on EMB medium have a green metallic sheen, Gram-stain negative, short rod, single, pair, or short chain. All isolates were identified as lactose fermenters with gas production, VP test negative, MR positive, motile test positive, citrate utilization negative, indole test positive, and H_2_S production negative. In addition to these tests, the PCR products of the *E. coli*-specific *phoA* gene of 65 bacterial strains, as shown by gel electrophoresis, included an amplified fragment of the 622-bp band, which was confirmed by the identification of these *E. coli* isolates by PCR. The results are presented in Figure-1.

### Isolation rate of APEC

Among the 65 chickens clinically diagnosed with colibacillosis, 65 APEC isolates were recovered from the 65 pools from different infected sites in the chicken.

### Phylotyping of APEC isolates using PCR

**Figure-2:**
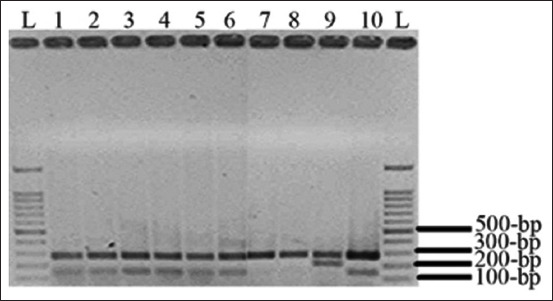
Phylogenetic groups of avian pathogenic *Escherichia coli* isolates recovered from broiler farms in Northern Palestine using Triplex polymerase chain reaction. Phylogenetic group D2 included lanes 1-6 and 10; Group D1 included lanes 7 and 8; Group B2 included lane 9; and lanes L comprised a 100-bp ladder.

Triplex PCR for phylogenetic classification showed that most APEC strains (56/65, 86.2%) belonged to group D. Other strains were assigned to groups B2 (5/65, 7.7%), B1 (3/65, 4.6%), and A (1/65, 1.5%). The results are presented in Table-3 and Figure-2. Statistical analyses showed a significant difference between the prevalence of the D group and the other groups at p < 0.05 using the Mann–Whitney U test. This indicated that the genetic markers (*chuA*, *yjaA*, and *TspE4.C2*) are differently distributed between the strains of the D group and those of the other groups.

**Table-3:** Occurrence of phylogenetic groups among 65 APEC isolates.

Group	Sub-group	Total number of APEC isolates (65)		

		No. of isolates	Sub-group %	Group %
A	A0	1	1.5	1.5
B1	B1	3	4.6	4.6
B2	B22	4	6.3	7.8
	B23	1	1.5	
D	D1	9	13.8	86.1
	D2	47	72.3	
Total		65	100	100

APEC=Avian pathogenic *Escherichia coli*

### Susceptibility of APEC isolates to antibiotics

**Figure-3:**
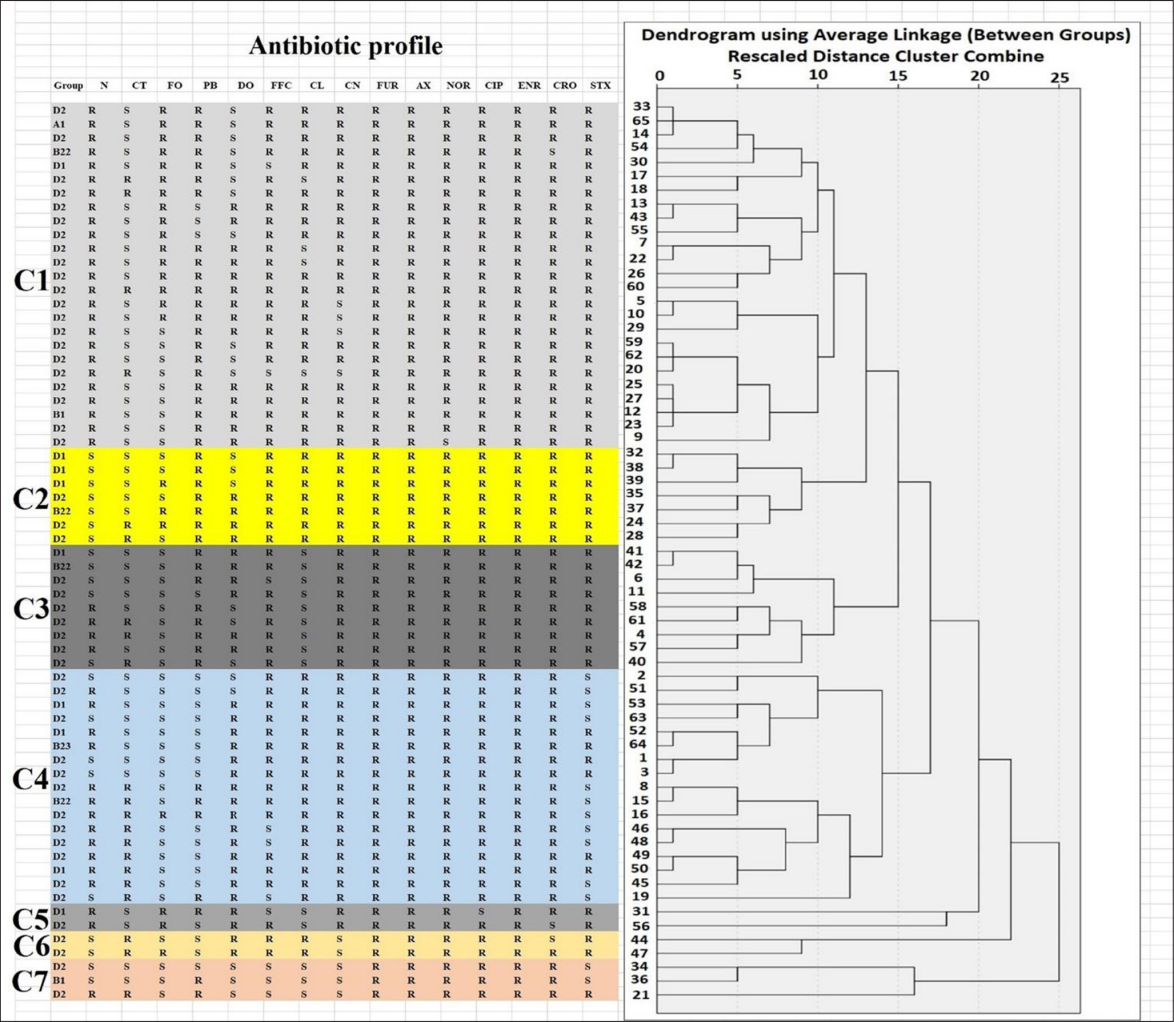
Dendrogram of 65 APEC strains isolated from broiler farms in Northern Palestine based on the UPGMA method derived from analysis of the antibiotic resistance profile and Phylogenetic groups. Intermediate antibiotic results for APEC isolates indicate resistance. APEC=Avian pathogenic *Escherichia coli*, N=Neomycin sulfate, CT=Colistin sulfate, PB=Polymyxin B, DO=Doxycycline, FFC=Florfenicol, CL=Cephalexin, CN=Gentamicin, FUR=Ceftiofur, AX=Amoxicillin, NOR=Norfloxacin, ENR=Enrofloxacin, CIP=Ciprofloxacin, CRO=Ceftriaxone, STX=Sulfamethoxazole/Trimethoprim, FO=Fosfomycin, C=Cluster.

The chemical structures of all tested antibiotics were categorized into nine distinct classes: aminopenicillin (AX), cephalosporines (CRO, CL, and FUR), tetracyclines (doxycycline), fluoroquinolones (CIP, NOR, and ENR), aminoglycosides (CN and neomycin), sulfonamides (trimethoprim/sulfamethoxazole), polymyxins (PB and polymyxin E [colistin]), amphenicols (FFC), and phosphonic antibiotic (FO). Antibiotic resistance ranged from 27.7% for polymyxin E (colistin) to 100% for AX. The results are presented in Table-4. The 65 APEC isolates revealed 48 resistance patterns, the most prevalent of which were neomycin sulfate, FO, PB, FFC, CL, CN, FUR, AX, NOR, CIP, ENR, CRO, and sulfamethoxazole/trimethoprim (STX) (Data not shown). This suggests that the APEC strains in these broiler farms in Northern Palestine exhibit various resistance patterns. Results showed that seven clusters depended on the resistance/sensitivity of the 65 APEC strains to the antibiotics used in this study, but the clustering process using a dendrogram was independent of phylogenetic groups. In addition, Clusters C1, C2, C3, and C4 can be divided into 2 or 3 sub-clusters. The results are shown in Figure-3. This number of clusters and sub-clusters confirms that the APEC strains in these broiler farms in Northern Palestine exhibit a wide variety of resistance patterns.

**Table-4:** The antibiotic resistance profile of 65 APEC isolates collected from broiler farms in northern Palestine.

Group	Antibiotic	Resistant strains	Intermediate strains	Susceptible strains
		
		No. of samples (%)	No. of samples (%)	No. of samples (%)
Tetracycline	Doxycycline	32 (49.2)	11 (16.9)	22 (33.8)
Fluoroquinolone	Ciprofloxacin	56 (86.2)	8 (12.3)	1 (1.5)
	Norfloxacin	58 (89.2)	6 (9.2)	1 (1.5)
	Enrofloxacin	64 (98.5)	1 (1.5)	0 (0.0)
Aminoglycoside	Gentamicin	52 (80.0)	5 (7.7)	8 (12.3)
	Neomycin	40 (61.5)	4 (6.2)	21 (32.3)
Sulfonamides	Sulfamethoxazole/trimethoprim	51 (78.5)	1 (1.5)	13 (20.0
Cephalosporins	Cephalexin	47 (72.3)	1 (1.5)	17 (26.2)
	Ceftriaxone	58 (89.2)	4 (6.2)	3 (4.6)
	Ceftiofur	60 (92.3)	5 (7.7)	0 (0.0)
Aminopenicillin	Amoxicillin	65 (100)	0 (0.0)	0 (0.0)
Polymyxins	Polymyxin E (colistin)	18 (27.7)	2 (3.0)	45 (69.2)
	Polymyxin B	36 (55.4)	8 (12.3)	21 (32.3)
Amphenicols	Florfenicol	55 (84.6)	1 (1.5)	9 (13.8)
Phosphonic antibiotic	Fosfomycin	23 (35.4)	0 (0)	42 (64.6)

APEC=Avian pathogenic *Escherichia coli*

Polymyxin E (colistin) and FO were the most effective drugs against these APEC isolates. The isolates from Group B1 were less resistant to the drug than those from Groups D and B2. An assessment was conducted on the association between the phylogenetic group and the mean antibiotic resistance score. The mean antibiotic resistance scores were 11, 11.8, and 8.7 for groups D, B2, and B1, respectively. A two-tailed t-test only showed a significant difference between the aggregate antibiotic resistance scores between isolates resistant to NOR and phylogenetic groups D and B1 in favor of group D. In addition, there was a significant difference between the aggregate antibiotic resistance scores between isolates resistant to CIP, NOR, and CL and phylogenetic groups B1 and B2 (p < 0.05), in favor of Group B2. The results are shown in Table-5. According to the prevalence of antibiotic resistance based on the phylogenetic groups, the results showed a significant difference between the prevalence of CL resistance between groups B1 and B2 (p < 0.05), in favor of the B2 group. The results are shown in Table-5.

**Table-5:** The antibiotic resistance between groups of 65 APEC isolates collected from broiler farms in Northern Palestine.

Group	Antibiotic	Resistant Strains n (%)	Distribution of AR according to phylogenetic groups n (%)			Aggregate AR score (mean)		
		
		n = 65 (%)	D n = 56 (%)	B1 n = 3 (%)	B2 n = 5 (%)	D AR score (mean) 618 (11.0)	B1 AR score (mean) 26 (8.7)	B2 AR score (mean) 59 (11.8)
Tetracycline	Doxycycline	32 (49.2)	30 (53.6)	1 (33.3)	1 (20.0)	342 (6.1)	11 (3.7)	13 (2.6)
Fluoroquinolone	Ciprofloxacin	56 (86.2)	48 (85.7)	2 (66.7)	5 (100)	542 (9.7)	16 (5.3)^a^	59 (11.8)^a^
	Norfloxacin	58 (89.2)	50 (89.3)	2 (66.7)	5 (100)	564 (10.1)^b^	16 (5.3)^b,c^	59 (11.8)^c^
	Enrofloxacin	64 (98.5)	55 (98.2)	3 (100)	5 (100)	613 (10.9)	26 (8.7)	59 (11.8)
Aminoglycoside	Gentamicin	52 (80.0)	44 (78.6)	2 (66.7)	5 (100)	504 (9)	21 (7.0)	59 (11.8)
	Neomycin	40 (61.5)	34 (60.7)	1 (33.3)	4 (80.0)	399 (7.1)	10 (3.3)	46 (9.2)
Sulfonamide	Sulfamethoxazole/trimethoprim	51 (78.5)	44 (78.6)	2 (66.7)	4 (80.0)	498 (8.9)	21 (7.0)	47 (9.4)
Cephalosporin	Cephalexin	47 (72.3)	40 (71.4)	1 (33.3)^e^	5 (100)^e^	542 (9.7)	10 (3.3)^d^	59 (11.8)^d^
	Ceftriaxone	58 (89.2)	51 (91.1)	3 (100)	4 (80.0)	571 (10.2)	26 (8.7)	47 (9.4)
	Ceftiofur	60 (92.3)	52 (92.9)	2 (66.7)	5 (100)	585 (10.4)	21 (7.0)	59 (11.8)
Aminopenicillin	Amoxicillin	65 (100.0)	56 (100.0)	3 (100)	5 (100)	618 (11.0)	26 (8.7)	59 (11.8)
Polymyxin	Polymyxins E (colistin)	18 (27.7)	17 (30.4)	0 (0.0)	1 (20.0)	200 (3.6)	0 (0.0)	12 (2.4)
	Polymyxin B	36 (55.4)	30 (53.6)	2 (66.7)	3 (60.0)	356 (6.4)	21 (7.0)	37 (7.4)
Amphenicol	Florfenicol	55 (84.6)	47 (83.9)	2 (66.7)	5 (100)	537 (9.6)	21 (7.0)	59 (11.8)
Phosphonic antibiotic	Fosfomycin	23 (35.4)	20 (35.7)	0 (0.0)	2 (40.0)	250 (4.5)	0 (0.0)	25 (5)

APEC=Avian pathogenic *Escherichia coli*, AR=Antibiotic resistance, The aggregate AR score (mean) and prevalence of AR between groups did not include isolates belonging to group A. ^a,b,c,d,e^Significant at p < 0.05 (t-test)

### Virulence genotyping of APEC isolates using PCR

**Figure-4:**
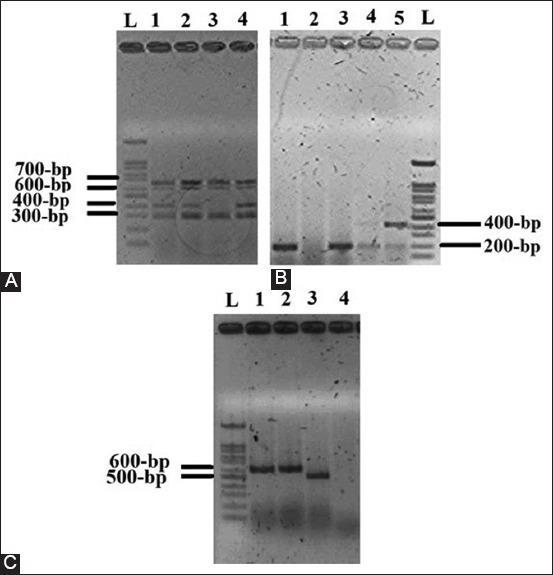
Multiplex polymerase chain reaction profiles specific to the avian pathogenic *Escherichia coli* virulence factors. Lane L has a 100-bp ladder. A: The genes *utaA* (302-bp), *tsh* (420-bp), *hlyF* (599-bp), and *iroN* (667-bp) are shown in Lanes 1, 2, 3, and 4. B: The *papGII* (190-bp) and *papGI* (461-bp) genes are shown in lanes 1, 3, 4, and 5.. C: The *papC* (501-bp) and *cva/cvi* (598-bp) are shown in lanes 1, 2, and 3.

**Figure-5:**
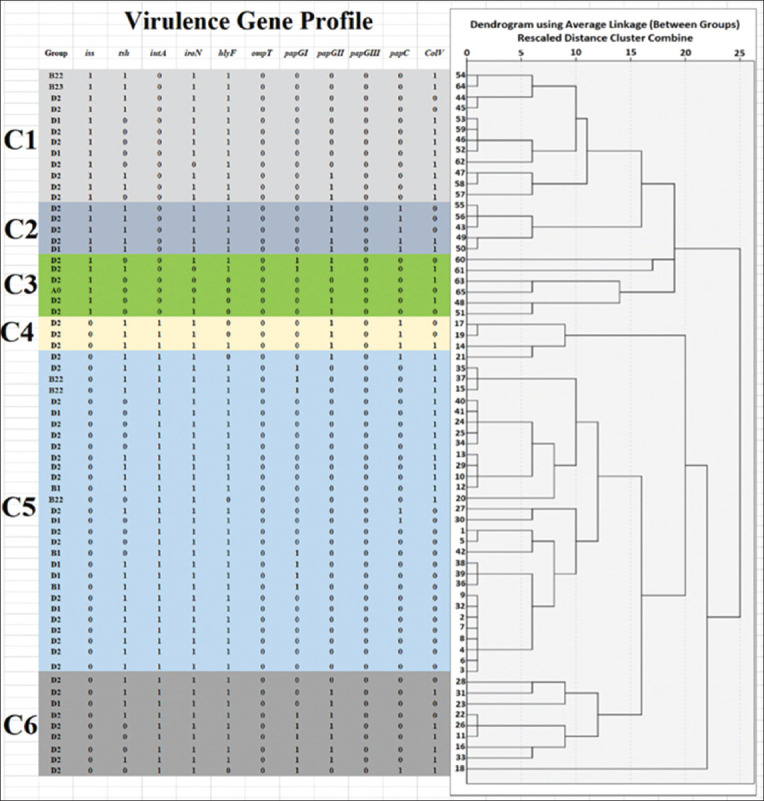
Dendrogram of 65 avian pathogenic *Escherichia coli* strains isolated from broiler farms in Northern Palestine based on the UPGMA method derived from analysis of the virulence factor profile and phylogenetic groups.

It was found that the most common virulence gene was *iroN*, which was detected in 61 isolates (93.8%). 56 (86.2%), 42 (64.6%), 40 (61.5%), 37 (56.9%), 24 (36.9%), 23 (35.4%),16 (24.6%), 13 (20.0%), 0 (0.0%), and 0 (0.0%) isolates were positive for *hlyF*, *iutA*, *Tsh*, *ColV*, *papGII*, *Iss*, *papGI*, *papC*, *papGIII*, and *ompT* genes, respectively. The results are shown in Figure-4 and Table-6. We found that 36 (55.4%) isolates had at least 5 genes. Thirty-four virulence gene patterns were observed in the 65 APEC isolates, with *Tsh*, *iutA*, *iroN*, and *hlyF* being the most predominant (12.3%). The results are shown in Table-7. Results showed that six clusters depend on the presence/absence of VFs in 65 APEC strains used in this study. The clustering process using a dendrogram is independent of phylogenetic groups. In addition, these clusters can be divided into 2 or more sub-clusters. The results are shown in Figure-5. This number of clusters and subclusters suggests that the APEC strains in these broiler farms in Northern Palestine exhibit a wide range of genetic variations.

**Table-6:** Virulence genes, virulence factor scores, prevalence of virulence factors, and their distribution to phylogenetic groups D, B1, and B2.

Virulence genes	Prevalence	Distribution of VFs according to phylogenetic groups			Aggregate VF score (mean)		
		
	n (%)	D n = 56 (%)	B1 n = 3 (%)	B2 n = 5 (%)	D n = 56 272 (4.86)	B1 n = 3 14 (4.67)	B2 n = 5 25 (5)
Adhesins genes							
*papGI*	16 (24.6)	12 (21.4)	2 (66.7)	2 (40.0)	65 (1.1)	9 (3.0)	12 (2.4)
*papGII*	24 (36.9)	24 (42.9)	0 (0.0)	0 (0.0)	140 (2.5)	0 (0.0)	0 (0.0)
*papGIII*	0 (0.0)	0 (0.0)	0 (0.0)	0 (0.0)	0 (0.0)	0 (0.0)	0 (0.0)
*papC*	13 (20.0)	13 (23.2)	0 (0.0)	0 (0.0)	77 (1.4)	0 (0.0)	0 (0.0)
*Tsh*	40 (61.5)	34 (60.7)	2 (66.7)	4 (80.0)	181 (3.2)	10 (3.3)	22 (4.4)
Iron acquisition system genes							
*iutA*	42 (64.6)	36 (64.3)	3 (100)	3 (60.0)	176 (3.1)	14 (4.7)	15 (3.0)
*iroN*	61 (93.8)	53 (94.6)	3 (100)	5 (100)	261 (4.7)	14 (4.7)	25 (5.0)
Cell protectin genes							
*iss*	23 (35.4)	20 (35.7)	0 (0.0)	2 (40.0)	96 (1.7)	0 (0.0)	10 (2.0)
*ompT*	0 (0.0)	0 (0.0)	0 (0.0)	0 (0.0)	0 (0.0)	0 (0.0)	0 (0)
Toxins genes							
*hlyF*	56 (86.2)	49 (87.5)	3 (100)	4 (80.0)	242 (4.3)	14 (4.7)	22 (4.4)
Colicin V operon							
*cva/cvi*	37 (56.9)	31 (55.4)	1 (33.3)*	5 (100)*	160 (2.9)	5 (1.7)	25 (5.0)

VF=Virulence factor, n=Number of isolates.

* Significant at p < 0.05

**Table-7:** Virulence gene patterns of 65 APEC isolates recovered from broiler farms in Northern Palestine.

Virulence gene patterns	No. of virulence gene patterns
*iutA, iroN, hlyF*	2
*Tsh, iutA, iroN, hlyF*	8
*Tsh, iutA, iroN, hlyF, cva/cvi*	4
*iutA, iroN, hlyF, papGI, papGII, cva/cvi*	3
*Tsh, iutA, iroN, hlyF, papGII, papC, cva/cvi*	1
*Tsh, iutA, iroN, hlyF, papGI, cva/cvi*	3
*Tsh, iutA, iroN, hlyF, papGI, papGII, cva/cvi*	1
*Tsh, iutA, iroN, papGII, papC*	2
*iutA, iroN,papGI, papC, cva/cvi*	1
*iutA, iroN, cva/cvi*	1
*Tsh, iutA, iroN, papGII, papC, cva/cvi*	1
*Tsh, iutA, iroN, hlyF, papGI, papGII*	1
*iutA, iroN, hlyF, cva/cvi*	5
*Tsh, iutA, iroN, hlyF, papC*	1
*Tsh, iutA, iroN, hlyF, papGII, cva/cvi*	1
*iutA, iroN, hlyF, papC*	1
*Tsh, iutA, iroN, hlyF, papGII*	1
*Tsh, iutA, iroN, hlyF, papGI, papGII, papC, cva/cvi*	1
*Tsh, iutA, iroN, hlyF, papGI*	3
*iutA, iroN, hlyF, papGI, papC*	1
*Iss, Tsh, iroN, hlyF, papGI, papC*	3
*Iss, Tsh, iroN, hlyF*	2
*Iss, iroN, hlyF, cva/cvi*	4
*Iss, Tsh, iroN, hlyF, papGII, cva/cvi*	2
*Iss,iroN, papGII, cva/cvi*	1
*Iss, Tsh, iroN, hlyF, papGII, papC, cva/cvi*	2
*Iss, iroN, papGII*	1
*Iss, tsh, iroN, hlyF, cva/cvi*	2
*Iss, iroN, hlyF, papGII, cva/cvi*	1
*Iss, iroN, hlyF, papGI, papGII*	1
*Iss, tsh, hlyF, papGI, papGII, cva/cvi*	1
*Iss, hlyF, cva/cvi*	1
*Iss, cva/cvi*	1
*Iss*	1
Total	65

APEC=Avian pathogenic *Escherichia coli*

A two-tailed t-test showed no significant difference between the aggregate VF scores of groups D, B1, and B2 (p < 0.05). According to the distribution of VFs based on the phylogenetic groups, the results showed a significant difference between the distribution of *cva/cvi* between groups B1 and B2 (p < 0.05), in favor of the B2 group. The results are presented in Table-6.

It was also found that *iroN* and *hlyF* genes were the most common genes among strains resistant to fluoroquinolones, cephalosporins, aminoglycoside, polymyxins, FO, doxycycline, florfenicol, and trimethoprim/sulfamethoxazole. The prevalence of *iroN* gene was 95.2%, 95.3%, 94.6%, 93.2%, 100%, 96.9%, 94.4%, and 96% for isolates resistant to fluoroquinolones, cephalosporins, aminoglycoside, polymyxins, FO, doxycycline, florfenicol, and trimethoprim/sulfamethoxazole, respectively. The prevalence of *hlyF* gene was 88.9%, 87.5%, 85.7%, 81.8%, 90.9%, 87.5%, 90.7%, and 92% for isolates resistant to fluoroquinolones, cephalosporins, aminoglycoside, polymyxins, FO, doxycycline, florfenicol, and trimethoprim/sulfamethoxazole, respectively. The *iutA* gene was the most common polymyxin-resistant strain. The results are shown in Tables-8–11.

**Table-8:** Virulence factors in relation to fluoroquinolones and cephalosporin-resistant phenotype among 64 APEC isolates recovered from broilers’ farms in Northern Palestine with colibacillosis.

VF	No. (%) of APEC isolates Fluoroquinolone-resistant and susceptible phenotype							

	Group D n (%)		Group B1 n (%)		Group B2 n (%)		Group D/B1/B2 n (%)	Group D/B1/B2 n (%)
				
	R (n= 55)	S (n=1)	R (n=3)	S (n=0)	R (n=5)	S (n= 0)	R (n=63)	S (n=1)
*papGI*	12 (21.8)	0 (0.0)	2 (66.7)	0 (0.0)	2 (40)	0 (0.0)	16 (25.4)	0 (0.0)
*papGII*	23 (41.8)	1 (100)	0 (0.0)	0 (0.0)	0 (0.0)	0 (0.0)	23 (36.5)	1 (100)
*papGIII*	0 (0.0)	0 (0.0)	0 (0.0)	0 (0.0)	0 (0.0)	0 (0.0)	0 (0.0)	0 (0.0)
*papC*	12 (21.8)	1 (100)	0 (0.0)	0 (0.0)	0 (0.0)	0 (0.0)	12 (19)	1 (100)
*Tsh*	34 (61.8)	0 (0.0)	2 (66.7)	0 (0.0)	4 (80)	0 (0.0)	40 (63.5)	0 (0.0)
*iutA*	35 (63.6)	1 (100)	3 (100)	0 (0.0)	3 (60)	0 (0.0)	41 (65.1)	1 (100)
*iroN*	52 (94.5)	1 (100)	3 (100)	0 (0.0)	5 (100)	0 (0.0)	60 (95.2)	1 (100)
*Iss*	20 (36.3)	0 (0.0)	0 (0.0)	0 (0.0)	2 (40)	0 (0.0)	22 (34.9)	0 (0.0)
*ompT*	0 (0.0)	0 (0.0)	0 (0.0)	0 (0.0)	0 (0.0)	0 (0.0)	0 (0.0)	0 (0.0)
*hlyF*	49 (89.1)	0 (0.0)	3 (100)	0 (0.0)	4 (80)	0 (0.0)	56 (88.9)	0 (0.0)
*cva/cvi*	30 (54.5)	1 (100)	1 (33.3)	0 (0.0)	5 (100)	0 (0.0)	36 (57)	1 (100)

**VF**	**No. (%) of APEC isolates Cephalosporin resistant and susceptible phenotype**							

	**Group D n (%)**		**Group B1 n (%)**		**Group B2 n (%)**		**Group D/B1/B2 n (%)**	**Group D/B1/B2 n (%)**
				
	**R (n = 56)**	**S (n = 0)**	**R (n = 3)**	**S (n = 0)**	**R (n = 5)**	**S (n = 0)**	**R (n = 64)**	**S (n = 0)**

*papGI*	12 (21.4)	0 (0.0)	2 (66.7)	0 (0.0)	2 (40)	0 (0.0)	16 (25)	0 (0.0)
*papGII*	24 (42.9)	0 (0.0)	0 (0.0)	0 (0.0)	0 (0.0)	0 (0.0)	24 (37.5)	0 (0.0)
*papGIII*	0 (0.0)	0 (0.0)	0 (0.0)	0 (0.0)	0 (0.0)	0 (0.0)	0 (0)	0 (0.0)
*papC*	13 (23.2)	0 (0.0)	0 (0.0)	0 (0.0)	0 (0.0)	0 (0.0)	13 (20.3)	0 (0.0)
*Tsh*	34 (60.7)	0 (0.0)	2 (66.7)	0 (0.0)	4 (80)	0 (0.0)	40 (62.5)	0 (0.0)
*iutA*	36 (64.3)	0 (0.0)	3 (100)	0 (0.0)	3 (60)	0 (0.0)	42 (65.6)	0 (0.0)
*iroN*	53 (94.6)	0 (0.0)	3 (100)	0 (0.0)	5 (100)	0 (0.0)	61 (95.3)	0 (0.0)
*Iss*	20 (35.7)	0 (0.0)	0 (0.0)	0 (0.0)	2 (40)	0 (0.0)	22 (34.4)	0 (0.0)
*ompT*	0 (0.0)	0 (0.0)	0 (0.0)	0 (0.0)	0 (0.0)	0 (0.0)	0 (0.0)	0 (0.0)
*hlyF*	49 (87.5)	0 (0.0)	3 (100)	0 (0.0)	4 (80)	0 (0.0)	56 (87.5)	0 (0.0)
*cva/cvi*	31 (55.4)	0 (0.0)	1 (33.3)	0 (0.0)	5 (100)	0 (0.0)	37 (57.8)	0 (0.0)

VF=Virulence factor, R=Resistant; S=Susceptible, APEC=Avian pathogenic *Escherichia coli*

**Table-9:** Virulence factors in relation to aminoglycoside and polymyxin-resistant phenotype among 64 APEC isolates recovered from broilers’ farms in Northern Palestine with colibacillosis.

VF	No. (%) of APEC isolates Aminoglycoside-resistant and susceptible phenotype							

	Group D n (%)		Group B1 n (%)		Group B2 n (%)		Group D/B1/B2 n (%)	Group D/B1/B2 n (%)
				
	R (n = 49)	S (n =7)	R (n = 2)	S (n = 1)	R (n = 5)	S (n = 0)	R (n = 56)	S (n = 8)
*papGI*	10 (20.4)	2 (28.6)	1 (50)	1 (100)	2 (40)	0 (0.0)	13 (23.2)	3 (37.5)
*papGII*	21 (42.9)	3 (42.9)	0 (0.0)	0 (0.0)	0 (0.0)	0 (0.0)	21 (37.5)	3 (37.5)
*papGIII*	0 (0.0)	0 (0.0)	0 (0.0)	0 (0.0)	0 (0.0)	0 (0.0)	0 (0.0)	0 (0.0)
*papC*	13 (26.5)	0 (0.0)	0 (0.0)	0 (0.0)	0 (0.0)	0 (0.0)	13 (20.3)	0 (0.0)
*Tsh*	29 (59.2)	5 (71.4)	1 (50)	1 (100)	4 (80)	0 (0.0)	34 (60.7)	6 (75)
*iutA*	31 (63.3)	5 (71.4)	2 100)	1 (0.0)	3 (60)	0 (0.0)	36 (64.3)	6 (75)
*iroN*	46 (93.9)	7 (100)	2 100)	1 (100)	5 (100)	0 (0.0)	53 (94.6)	8 (100)
*Iss*	18 (36.7)	2 (28.6)	0 (0.0)	0 (0.0)	2 (40)	0 (0.0)	20 (35.7)	2 (25)
*ompT*	0 (0.0)	0 (0.0)	0 (0.0)	0 (0.0)	0 (0.0)	0 (0.0)	0 (0.0)	0 (0.0)
*hlyF*	42 (85.7)	7 (100)	2 100)	1 (100)	4 (80)	0 (0.0)	48 (85.7)	8 (100)
*cva/cvi*	28 (57.1)	3 (42.9)	1 (50)	0 (0.0)	5 (100)	0 (0.0)	34 (60.7)	3 (37.5)

**VF**	**No. (%) of APEC isolates Polymyxins resistant and susceptible phenotype**							

	**Group D n (%)**		**Group B1 n (%)**		**Group B2 n (%)**		**Group D/B1/B2 n (%)**	**Group D/B1/B2 n (%)**
				
	**R (n = 38)**	**S (n = 18)**	**R (n = 2)**	**S (n = 1)**	**R (n = 3)**	**S (n = 2)**	**R (n = 44)**	**S (n = 20)**

*papGI*	10 (26.3)	2 (11.1)	1 (50)	1 (100)	2 (66.7)	0 (0.0)	13 (29.5)	3 (15)
*papGII*	21 (55.2)*	3 (16.7)*	0 (0.0)	0 (0.0)	0 (0.0)	0 (0.0)	21 (47.7)*	3 (15)*
*papGIII*	0 (0.0)	0 (0.0)	0 (0.0)	0 (0.0)	0 (0.0)	0 (0.0)	0 (0.0)	0 (0.0)
*papC*	13 (34.2)	0 (0.0)	0 (0.0)	0 (0.0)	0 (0.0)	0 (0.0)	13 (29.5)	0 (0.0)
*Tsh*	29 (76.3)*	5 (27.8)*	1 (50)	1 (100)	3 (100)	1 (50)	33 (75)*	7 (35)*
*iutA*	31 (81.6)*	5 (27.8)*	2 (100)	1 (100)	2 (66.7)	1 (50)	36 (81.8)*^∞^	6 (30)*^∞^
*iroN*	36 (94.7)	17 (94.4)	2 (100)	1 (100)	3 (100)	2 (100)	41 (93.2)	20 (100)
*Iss*	18 (47.4)*	2 (11.1)*	0 (0.0)	0 (0.0)	2 (66.7)	0 (0.0)	20 (45.4)*^∞^	2 (10)*^∞^
*ompT*	0 (0.0)	0 (0.0)	0 (0.0)	0 (0.0)	0 (0.0)	0 (0.0)	0 (0.0)	0 (0.0)
*hlyF*	34 (89.5)	15 (83.3)	2 (100)	1 (100)	3 (100)	1 (50)	39 (81.8)	17 (85)
*cva/cvi*	21 (55.3)	10 (55.6)	1 (50)	0 (0.0)	3 (100)	2 (100)	25 (56.8)	12 (60)

VF=Virulence factor, R=Resistant, S=Susceptible, APEC=Avian pathogenic *Escherichia coli*,

∞ Significant at p < 0.05 (Chi-square statistic),

* Significant at p < 0.05 (t-test)

**Table-10:** Virulence factors in relation to the fosfomycin- and doxycycline-resistant phenotype among 64 APEC isolates recovered from broilers’ farms in Northern Palestine with colibacillosis.

VF gene	No. (%) of APEC isolates Fosfomycin-resistant and susceptible phenotype							

	Group D n (%)		Group B1 n (%)		Group B2 n (%)		Group D/B1/B2 n (%)	Group D/B1/B2 n (%)
		
	R (n = 20)	S (n = 36)	R (n = 0)	S (n = 3)	R (n = 2)	S (n = 3)	R (n = 22)	S (n = 42)
*papGI*	7 (35)	5 (13.9)	0 (0)	2 (66.7)	1 (50)	1 (33.3)	8 (36.4)	8 (11.9)
*papGII*	12 (60)	12 (33.3)	0 (0)	0 (0)	0 (0)	0 (0)	12 (54.5)*	12 (28.6)*
*papGIII*	0 (0)	0 (0)	0 (0)	0 (0)	0 (0)	0 (0)	0 (0)	0 (0)
*papC*	8 (40)*	5 (13.9)*	0 (0)	0 (0)	0 (0)	0 (0)	8 (36.4)*	5 (11.9)*
*Tsh*	13 (65)	21 (58.3)	0 (0)	2 (66.7)	2 (100)	2 (66.7)	15 (68.2)	25 (59.5)
*iutA*	15 (75)	21 (58.3)	0 (0)	3 (100)	1 (50)	2 (66.7)	16 (72.7)	26 (61.9)
*iroN*	20 (100)	33 (91.7)	0 (0)	3 (100)	2 (100)	3 (100)	22 (100)	39 (92.9)
*Iss*	5 (25)	15 (41.7)	0 (0)	0 (0)	1 (50)	1 (33.3)	6 (27.3)	16 (38.1)
*ompT*	0 (0)	0 (0)	0 (0)	0 (0)	0 (0)	0 (0)	0 (0)	0 (0)
*hlyF*	18 (90)	31 (86.1)	0 (0)	3 (100)	2 (100)	2 (66.7)	20 (90.9)	36 (85.7)
*cva/cvi*	9 (45)	22 (61.1)	0 (0)	1 (33.3)	2 (100)	3 (100)	11 (50)	26 (61.9)

**VF**	**No. (%) of APEC isolates Doxycycline-resistant and susceptible phenotype**							

	**Group D n (%)**		**Group B1 n (%)**	**Group B2 n (%)**	**Group D/B1/B2 n (%)**	**Group D/B1/B2 n (%)**
			
	**R (n = 30)**	**S (n = 26)**	**R (n = 1)**	**S (n =2)**	**R (n = 1)**	**S (n = 4)**	**R (n = 32)**	**S (n = 32)**
*papGI*	6 (20)	6 (23.1)	1 (100)	1 (50)	1 (100)	1 (25)	8 (25)	8 (25)
*papGII*	13 (60)	11 (60)	0 (0)	0 (0)	0 (0)	0 (0)	13 (40.6)	11 (34.4)
*papGIII*	0 (0)	0 (0)	0 (0)	0 (0)	0 (0)	0 (0)	0 (0)	0 (0)
*papC*	4 (13.3)	9 (34.6)	0 (0)	0 (0)	0 (0)	0 (0)	4 (12.5)	9 (28.1)
*Tsh*	18 (60)	16 (61.5)	0 (0)	2 (100)	1 (100)	3 (75)	19 (59.4)	21 (65.6)
*iutA*	20 (66.7)	16 (61.5)	1 (100)	2 (100)	1 (100)	2 (50)	22 (68.8)	20 (62.5)
*iroN*	29 (96.7)	24 (92.3)	1 (100)	2 (100)	1 (100)	4 (100)	31 (96.9)	30 (93.8)
*Iss*	10 (33.3)	10 (38.5)	0 (0)	0 (0)	0 (0)	2 (33.3)	10 (31.3)	12 (37.5)
*ompT*	0 (0)	0 (0)	0 (0)	0 (0)	0 (0)	0 (0)	0 (0)	0 (0)
*hlyF*	27 (85.7)	22 (100)	0 (0)	3 (100)	1 (100)	3 (75)	28 (87.5)	28 (87.5)
*cva/cvi*	18 (60)	13 (50)	0 (0)	1 (50)	1 (100)	4 (100)	19 (59.4)	18 (56.3)

APEC=Avian pathogenic *Escherichia coli*, VF=Virulence factor, R=Resistant, S=Susceptible.

* Significant at p < 0.05 (t-test)

**Table-11:** Virulence factors in relation to the florfenicol and sulfamethoxazole/trimethoprim-resistant phenotype among 64 APEC isolates recovered from broilers’ farms in Northern Palestine with colibacillosis.

VF	No. (%) of APEC isolates Florfenicol-resistant and susceptible phenotype							

	Group D n (%)		Group B1 n (%)		Group B2 n (%)		Group D/B1/B2 n (%)	Group D/B1/B2 n (%)
				
	R (n = 47)	S (n = 9)	R (n = 2)	S (n = 1)	R (n = 5)	S (n = 0)	R (n = 54)	S (n = 10)
*papGI*	11 (23.4)	1 (11.1)	1 (50)	1 (100)	2 (40)	0 (0.0)	14 (25.9)	2 (20)
*papGII*	19 (40.4)	5 (45)	0 (0.0)	0 (0.0)	0 (0.0)	0 (0.0)	19 (35.2)	5 (50)
*papGIII*	0 (0.0)	0 (0.0)	0 (0.0)	0 (0.0)	0 (0.0)	0 (0.0)	0 (0.0)	0 (0.0)
*papC*	10 (21.3)	3 (33.3)	0 (0.0)	0 (0.0)	0 (0.0)	0 (0.0)	10 (18.5)	3 (30)
*Tsh*	30 (63.8)	4 (44.4)	1 (50)	1 (100)	4 (80)	0 (0.0)	35 (64.8)	5 (50)
*iutA*	29 (61.7)	7 (77.8)	2 (100)	1 (100)	3 (60)	0 (0.0)	34 (63)	8 (80)
*iroN*	44 (93.6)	9 (100)	2 (100)	1 (100)	5 (100)	0 (0.0)	51 (94.4)	10 (100)
*Iss*	18 (38.3)	2 (2.2)	0 (0.0)	0 (0.0)	2 (40)	0 (0.0)	20 (37)	2 (20)
*ompT*	0 (0.0)	0 (0.0)	0 (0.0)	0 (0.0)	0 (0.0)	0 (0.0)	0 (0.0)	0 (0.0)
*hlyF*	43 (91.5)*	6 (66.7)*	2 (100)	1 (100)	4 (80)	0 (0.0)	49 (90.7)	7 (70)
*cva/cvi*	26 (55.3)	5 (55.6)	1 (50)	0 (0.0)	5 (100)	0 (0.0)	32 (59.3)	5 (50)

**VF**	**No. (%) of APEC isolates Sulfamethoxazole/trimethoprim resistant and Susceptible phenotype**							

	**Group D n (%)**		**Group B1 n (%)**		**Group B2 n (%)**		**Group D/B1/B2 n (%)**	**Group D/B1/B2 n (%)**
				
	**R (n = 44)**	**S (n = 12)**	**R (n = 2)**	**S (n = 1)**	**R (n = 4)**	**S (n = 1)**	**R (n = 50)**	**S (n = 14)**

*papGI*	10 (22.7)	2 (16.7)	1 (50)	1 (100)	1 (25)	1 (100)	12 (24)	4 (28.6)
*papGII*	20 (45.5)	4 (33.3)	0 (0.0)	0 (0.0)	0 (0.0)	0 (0.0)	20 (40)	4 (28.6)
*papGIII*	0 (0.0)	0 (0.0)	0 (0.0)	0 (0.0)	0 (0.0)	0 (0.0)	0 (0.0)	0 (0.0)
*papC*	11 (25)	2 (16.7)	0 (0.0)	0 (0.0)	0 (0.0)	0 (0.0)	11 (22)	2 (14.3)
*Tsh*	28 (63.6)	6 (50)	1 (50)	1 (100)	3 (75)	1 (100)	32 (64)	8 (57.1)
*iutA*	30 (68.2)	6 (50)	2 (100)	1 (100)	2 (50)	1 (100)	34 (68)	8 (57.1)
*iroN*	42 (95.5)	11 (68.8)	2 (100)	1 (100)	4 (100)	1 (100)	48 (96)	13 (92.9)
*Iss*	14 (31.8)	6 (50)	0 (0.0)	0 (0.0)	2 (50)	0 (0.0)	16 (32)	6 (42.9)
*ompT*	0 (0.0)	0 (0)	0 (0.0)	0 (0.0)	0 (0.0)	0 (0.0)	0 (0.0)	0 (0.0)
*hlyF*	41 (93.2)*	8 (66.7)*	2 (100)	1 (100)	3 (75)	1 (100)	46 (92)*	10 (71.4)*
*cva/cvi*	25 (56.8)	6 (50)	1 (50)	0 (0)	4 (100)	1 (100)	30 (60)	7 (50)

APEC=Avian pathogenic *Escherichia coli*, VF=Virulence factor, R=Resistant, S=Susceptible,

* Significant at p < 0.05 (t-test)

This study detected significant associations (p < 0.05) between specific viral factor levels and phenotypic resistance among clinical APEC isolates. A significant association was shown between *iutA* and *iss* VFs with polymyxins-resistant phenotype isolates. The results are presented in Table-9.

In addition, a two-tailed t-test showed that *papGII*, *Tsh*, *iutA*, and *iss* VFs were more prevalent in isolates that showed the polymyxin-resistant phenotype than in sensitive isolates, as well as more common in polymyxin-resistant phenotype isolates that belonged to Group D than in sensitive isolates in the same group (p < 0.05). The results are presented in Table-9. The same *papGII* and *papC* VFs were more prevalent in isolates that exhibited the FO-resistant phenotype than in sensitive isolates. The *papC* VF was more common in FO-resistant phenotype isolates belonging to Group D than in sensitive isolates in the same group (p < 0.05). The results are shown in Table-10.

Furthermore, the *hlyF* VF was more common in the FFC- and STX-resistant phenotype isolates that belonged to Group D than in the sensitive isolates in the same group (p < 0.05). However, *the hlyF* VF was more common in isolates that exhibited the STX-resistant phenotype regardless of the type of phylogenetic group (p < 0.05). The results are shown in Table-11.

## Discussion

Because of its diverse DNA, *E. coli* can grow in various hosts and settings. These bacteria can survive as pathogens or commensal organisms because they have evolved alongside humans and colonized various hosts [26].

The broiler industry faces serious economic and welfare problems because of APEC. More recent studies have highlighted the critical role of broiler breeders as APEC infection reservoirs through vertical transmission to chickens and subsequent horizontal transmission between chickens. Furthermore, all poultry species must be continuously tracked and monitored for APEC strains because of their continually evolving genetic diversity [9]. In addition, the detection of APEC isolates as a foodborne pathogen that contaminates chickens during meat processing and can be transmitted to consumers at risk. Therefore, studies on the genetic diversity of APEC isolates recovered from infected chickens obtained from broiler farms in Palestine could be used to reduce consumer risk, economic losses due to chicken mortality, decreased egg and meat production, and antibiotic therapy expenses.

Results showed a high detection rate (100%), similar to those of previously published studies. The detection rates of APEC strains ranged from 77.0% to 90.4% in Nepal [3, 27, 28], 100% in India [29], and 86.7% in Algeria [30].

The APEC strains isolated from poultry in this study belonged to phylogenetic group D. ExPEC, including APEC, is classified as belonging to phylogroup B2 and D, according to several published studies [9, 26, 31]. Through phylogenic examinations, this *E. coli* was found to have a noticeable overlap with the phylogroup of *E. coli* that infects people. This overlap suggests a significant zoonotic potential for APEC, which may result from parallel evolution and development in both hosts from a shared parent lineage [26]. These findings correspond with previous studies by Kim *et al*. [7] and Thomrongsuwannakij *et al*. [8] on APEC phylogenetic groupings, as it was reported that 41.1%–46.8% of common APEC isolates recovered from commercial broilers belonged to Group D. A study conducted in Palestine showed that UPEC isolates recovered from human urine indicated that 72.0% of the isolates belonged to phylogenetic Group D [32].

The findings of this study contradict those of another study concerning phylogenetic groups in APEC, which showed that most APEC isolates of colibacillosis recovered from broiler breeders belonged to either phylogenetic Group A, B1, or B2 [6–9, 12].

Antimicrobial growth promoter supplementation is the main strategy used to prevent APEC infections. Antimicrobial resistance among APEC isolates has increased dramatically worldwide [5–8, 10, 11]. This study detected a high resistance rate to AX, ENR, FUR, CRO, NOR, florfenicol, CIP, CN, STX, and CL. These antimicrobials are most commonly used in animals and poultry, which might explain the high resistance of isolates to these antibiotics. These results are similar to the findings of previous studies conducted in Pakistan [5], China [6], Qatar [10], and Bangladesh [11].

In this study, 58 resistance patterns were observed in the 65 APEC isolates and 7 clusters depended on the resistance/sensitivity of 65 APEC strains to the antibiotics used in this study; however, the clustering process using a dendrogram is independent of phylogenetic groups. In addition, clusters C1, C2, C3, and C4 can be divided into 2 or 3 sub-clusters, indicating a high diversity of resistance patterns in APEC strains in these broilers’ farms in Northern Palestine. These results also showed that all APEC isolates were MDR. The results of this study are in agreement with those of other studies by Azam *et al*. [5], Afayibo *et al*. [6], Ievy *et al*. [11], and Hussein *et al*. [12], which showed that all or most of the examined isolates were MDR. This may be due to the extensive and inappropriate use of antimicrobial drugs, such as overuse, prophylactic use, feed additives, or growth promoter use, which may contribute to the rise in antimicrobial resistance [8, 9]. The use of antibiotics in the production of animals for food is not restricted in Palestine. Antimicrobial resistance in bacterial populations is associated with the extensive use of antibiotics for growth enhancement in animal feed production. Antimicrobial resistance in humans and the use of antibiotics in animal feed production are closely associated in several studies. Antibiotic misuse in livestock threatens the clinical utility of antibiotics in humans and animals. It would be preferable to control avian colibacillosis through vaccination [3].

Plasmids are the primary source of VFs that contribute to the bacterial characteristics required to establish an APEC infection. Therefore, it is frequently not possible to identify APECs using phylogenetic classification according to chromosomal markers. Furthermore, the relationship between phylogenetic taxonomy and pathotype is more complicated because *E. coli* can acquire virulent plasmids from other bacteria [26]. The functions of these genes may be necessary for the development of APEC infections in poultry to develop. As a result, APECs may be differentiated from commensal, intestinal, environmental, and other extraintestinal *E. coli* strains using these genes. Certain plasmid-carried virulence-associated genes, including *hlyF*, *ompT*, *iroN*, *iss*, and *iutA*, are often present in APEC strains. These genes can be used to differentiate nonpathogenic *E. coli* strains from APEC strains [18]. The most frequently occurring virulence genes among the APEC isolates in the current study were those encoding iron acquisition genes *iroN* (93.8%) and *iutA* (64.6%) and toxins *hlyF* (86.2%). These genes are more frequently related to highly pathogenic APEC and are associated with the ColV plasmid, indicating that these isolates harbor plasmid PAIs [9, 12, 25]. These findings are similar to the results of previous studies conducted in China [6], Korea [7], USA [9, 26], Qatar [10], Egypt [12], Italy [13], and Australia [33].

APEC isolates must be characterized to fully understand the pathogenesis of colibacillosis and develop effective preventive and control measures. Results of this study suggest that PAI is not most frequently found on ColV plasmids in APEC isolates but might occur in other genomic locations. It is not necessary to have the 5 genes (*iroN*, *ompT*, *hlyF*, *iss*, and *iutA*) together. PAIs can be observed in APEC isolates at alternative sites, such as on ColBM plasmids [22]. Diagnostic approaches for recognizing APEC isolates are based on identifying several *E. coli* virulence genes [5]. According to Johar *et al*. [10], the APEC strain is considered pathogenic if it contains more than 4 of the detected genes. Based on this, only 55.4% of the strains in this study were considered pathogenic. The results of this study are consistent with that reported by Azam *et al*. [5], which showed that 42.6% and 56% of isolates were considered virulent APEC [13], based on the genetic criteria of possessing more than 4 VFs. However, compared with these three ColV plasmid genes (*iroN*, i*utA*, and *hlyF*), the gene *cva/cvi*, which is part of the ColV plasmid, was shown to have a lower prevalence. Although this was unusual, it has also been reported by Joseph *et al*. [9] and De Oliveira *et al*. [34].

Six clusters were observed and depended on the presence/absence of VFs in 65 APEC strains used in this study: each can be divided into 2 or more sub-clusters. These results indicate that these isolates have a high diversity of virulence genes. Information about virulence genes can be used to diagnose colibacillosis and identify vaccine candidates.

The *iutA* gene was the most common polymyxin-resistant strain. There was a significant association (p < 0.05) between specific VFs and phenotypic resistance among clinical APEC isolates. A significant association was found between *iutA* and *iss* VFs with polymyxins-resistant phenotype isolates. According to Awawdeh *et al*. [33], significant associations (p < 0.05) between specific virulence gene content and phenotypic resistance were detected among clinical APEC isolates. These included *iroN* with ampicillin resistance and *iss*-*iutA*-*ompT*-*hlyF*-*iroN* with CN resistance for clinical APEC isolates.

According to the distribution of VFs based on the phylogenetic groups, the results showed that the B2 group (100%) had a higher prevalence of *cva/cvi* VF than Group B1 (33.3%) (p < 0.05). In a recent study, certain phylotypes were associated with certain VFs, as demonstrated by the finding that groups B1 and D had a higher prevalence of *papC* and *vat* VFs than Groups A and B2. The virulence determinants *fyuA*, *iucD*, and *cva/cvi* were widely distributed in Groups D (84.78%), B1 (80.95%), and A (80.65%), respectively. Furthermore, *neuC* and *vat* expression was lower in Group A strains, although phylotype A strains mainly expressed *ibeA* [6].

## Conclusion

These results serve as an outline for further investigation into the pathophysiology of APEC and the development of efficient intervention plans for the prevention and management of APEC in broiler breeders. Furthermore, we conclude that polymyxin E (colistin) and FO should be the first antimicrobial agents of choice to combat infections caused by APEC in Palestine. The determination of the virulence genes of APEC strains and antibiotic resistance is essential in understanding their pathogenesis, antimicrobial therapy, and the future development of measures to control colibacillosis, such as vaccination, feed hygiene, and housing management strategies.

Maintaining control of APEC infections is essential for public health, especially when MDR genes are present in APEC isolates. In addition, APEC isolates can pass on resistance and virulence genes to dangerous bacteria, such as *E. coli*, which are specific to humans. Thus, the expansion of this study to include people working in the broilers’ farms is necessary to further evaluate the significance of APEC strains in human disease. In addition, it is essential to increase the sample size as well as the time duration of sample collection.

## Authors’ Contributions

GA, SA, and MA: Conceptualization, methodology, investigation, and data curation. GO: Statistical analyses and drafted and revised the manuscript. All authors have read and approved the final manuscript.
